# MicroRNAs miR-17 and miR-20a Inhibit T Cell Activation Genes and Are Under-Expressed in MS Whole Blood

**DOI:** 10.1371/journal.pone.0012132

**Published:** 2010-08-11

**Authors:** Mathew B. Cox, Murray J. Cairns, Kaushal S. Gandhi, Adam P. Carroll, Sophia Moscovis, Graeme J. Stewart, Simon Broadley, Rodney J. Scott, David R. Booth, Jeannette Lechner-Scott, ANZgene Multiple Sclerosis Genetics Consortium

**Affiliations:** 1 Hunter Medical Research Institute, The University of Newcastle, Newcastle, New South Wales, Australia; 2 Schizophrenia Research Institute, Sydney, New South Wales, Australia; 3 Westmead Millennium Institute, University of Sydney, Sydney, New South Wales, Australia; 4 Department of Neurology, Griffith University, Gold Coast, Queensland, Australia; National Institutes of Health, United States of America

## Abstract

It is well established that Multiple Sclerosis (MS) is an immune mediated disease. Little is known about what drives the differential control of the immune system in MS patients compared to unaffected individuals. MicroRNAs (miRNAs) are small non-coding nucleic acids that are involved in the control of gene expression. Their potential role in T cell activation and neurodegenerative disease has recently been recognised and they are therefore excellent candidates for further studies in MS. We investigated the transcriptome of currently known miRNAs using miRNA microarray analysis in peripheral blood samples of 59 treatment naïve MS patients and 37 controls. Of these 59, 18 had a primary progressive, 17 a secondary progressive and 24 a relapsing remitting disease course. In all MS subtypes miR-17 and miR-20a were significantly under-expressed in MS, confirmed by RT-PCR. We demonstrate that these miRNAs modulate T cell activation genes in a knock-in and knock-down T cell model. The same T cell activation genes are also up-regulated in MS whole blood mRNA, suggesting these miRNAs or their analogues may provide useful targets for new therapeutic approaches.

## Introduction

Multiple Sclerosis (MS) is a disease which affects mainly young people that often leaves them disabled in their most productive years. It is a relatively common disease with an incidence somewhere between 1–2 per 1000 and the rate appears to be increasing [1]. It is assumed that a T cell dysregulation results in demyelination and axonal loss throughout the central nervous system [2]. Most patients have a relapsing remitting course (RRMS), which is unpredictable and is observed as episodes of acute inflammation that results in neurological dysfunction, which in the majority of cases responds to immunomodulatory steroid treatment. Relapsing remitting disease is characterized by some level of myelin repair, whereas in the progressive form myelin repair seems to be insufficient or ineffectual resulting in progressive disability without any observable signs of recovery.

In recent years genome wide association studies have identified that not only is there an association with haplotype in the Human Leukocyte Antigen (HLA) region, but also in both the *IL-2* and the *IL-7* receptor genes, *CD56*, *CD226* and *CLEC16A*, which together are considered to contribute to a predisposition to develop the disease [3]–[5].

In the largest single genome wide association study searching for genetic risk factors for MS, polymorphisms associated with the disease confirmed the importance of immune dysfunction [5]. These identified polymorphisms alone or in combination do not explain the significant differences in immune function associated with this disease.

MicroRNAs (miRNAs) have recently been discovered to be regulatory modulators of gene expression [6]. A striking feature of these mRNA regulators is their evolutionary conservation, indicating their level of importance in the control of gene expression [7]. MiRNAs bind to the 3′ UTR of target mRNA through base pairing, resulting in target mRNA cleavage or translation inhibition [8]. On average each miRNA regulates about 200 genes, and the outcome of regulation is cell state and type specific [9]. Their dysregulation has been associated with many diseases, and the potential for modulating their action by therapeutic intervention has excited much interest [10].

This is particularly so in immune-related diseases. The miRNA transcriptome of immune cell subsets are distinct, suggesting that naïve, effector and central memory T cell [11] and regulatory T cell [12] function is dependent on the miRNA regulation. Gross changes to mouse miRNA regulation by deletion of the genes which mediate it, Dicer and Drosher, results in T cell abnormalities and autoimmune disease [13]. Autoimmune diseases such as MS are ameliorated by drugs modulating T cell function like interferon beta and glatiramer acetate, and with monoclonal antibodies specific to T cell surface markers [14]. The genes which are associated with this, and other autoimmune diseases, are predominately expressed in antigen presenting cells and T cells, and there is a marked T cell activation gene expression pattern in MS whole blood [15]–[17]. One of the difficulties of studying multiple sclerosis is the acquisition of samples unaffected by the influence of immunomodulatory treatment. In this report we have investigated miRNA expression profiles of a series of effectively treatment naïve MS patients (i.e. all patients were free of disease modifying therapy for at least 3 months) compared to a healthy age-matched control group. The findings of this study demonstrate the differences between the immune function in MS without the influence of any treatment regiment.

## Results

We measured the known miRNA transcriptome in whole blood from 59 MS patients and 37 healthy controls using the Illumina sentrix array matrix. Of these 59 patients, 18 had a primary progressive (PPMS), 17 a secondary progressive (SPMS) and 24 a relapsing remitting course. The patient demographics are shown in table S1. To ensure we were not measuring effects due to diurnal variation in immune function, all samples were collected between the hours of 9am–1pm for both the MS and healthy control populations. All of our patients were of Caucasian origin, and had not received any immunomodulatory therapy within the previous 3 months. The female to male ratio was 2∶1 similar to the disease incidence in the general MS population. The age range for the entire group was 32 to 81 years with a mean age of 54 years, mean time since diagnosis was 20 years, ranging from 0 to 58 years, mean expanded disability status scale (EDSS) was 4.5 ranging from 0 to 6. There was no significant difference in the demographics between the group assessed by microarray and the one assessed by RT-PCR. As expected the disease duration and the EDSS was higher in the SP and PP group compared to the RR group.

**Figure 1:**
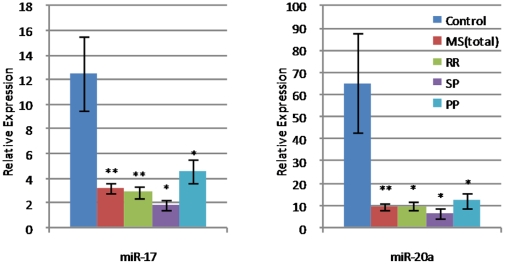
Relative expression of target miRNA. Relative expression of target miRNA compared to endogenous control miRNA in MS subtypes and healthy controls, validation by Q-RTPCR. The two miRNAs were significantly under-expressed in MS patients compared to healthy controls (*P<0.05, **P<0.01), error bars ± SEM.

MiRNA expression analysis revealed that out of 733 miRNAs assessed, 26 were down-regulated and 1 up-regulated in MS whole blood, based on a false discovery rate of less than 1% (Table 1). The fold change ranged between 1.36 and −1.59. The down-regulation of miRNAs was across all the subtypes of MS. Out of the 27 we focused on miR-17 and miR-20a as the differential expression analysis revealed that these miRNAs were the most significantly different in the MS group compared to the controls. Both miRNAs are known to be involved in immune function and their importance was confirmed by RT-PCR performed between 3 and 6 times. Both miRNAs were differently expressed across all MS subtypes (Figure 1). Most significantly different was miR-17 with a p-value of 7.61 e-05 (see Table 2).

**Table 1:** miRNAs dysregulated in MS whole blood.

Gene Name	Fold-Change	D Value
hsa-miR-768-3p:11.0	1.36	3.81
HS_265.1	−1.13	−3.06
hsa-let-7d	−1.14	−2.85
hsa-let-7f	−1.34	−3.81
hsa-let-7g	−1.19	−3.66
hsa-let-7i	−1.11	−3.27
hsa-miR-106a:9.1	−1.3	−4.09
hsa-miR-126	−1.22	−3.68
hsa-miR-126*	−1.51	−3.66
hsa-miR-140-5p	−1.32	−2.87
hsa-miR-15a	−1.12	−2.89
hsa-miR-15b	−1.07	−3.14
hsa-miR-16	−1.08	−3.18
hsa-miR-17	−1.59	−4.72
hsa-miR-20a	−1.18	−4.44
hsa-miR-20b	−1.25	−3.31
hsa-miR-211	−1.09	−2.92
hsa-miR-27a	−1.32	−3.27
hsa-miR-27b	−1.26	−3.47
hsa-miR-374a	−1.26	−4.27
hsa-miR-454	−1.54	−3.94
hsa-miR-510	−1.13	−2.98
hsa-miR-579	−1.18	−2.77
hsa-miR-623	−1.17	−2.95
hsa-miR-624*	−1.25	−2.83
hsa-miR-93	−1.08	−3.72
hsa-miR-98	−1.49	−4.18

**Table 2:** P values of relative expression of target miRNAs.

	miR-17	miR-20a
**MS_(total)_**	7.61E-05	9.43E-04
**RR**	4.21E-03	1.95E-02
**SP**	1.39E-02	5.00E-02
**PP**	3.20E-02	4.68E-02

P-values of the relative expression as determined by Q-RTPCR of miR-17 and miR-20a in MS patients as a whole and their respective subgroups compared against a healthy aged-matched population.

To better define the effects of miR-17 and miR-20a on gene expression we conducted knock-in (with synthetic miRNA) and knock-down (with LNA modified anitmiRs) experiments using Jurkat cells (performed in triplicate). Genes down-regulated by the miR-17 transformants, and up-regulated by the transformants of small interfering RNAs (siRNAs) corresponding to it were identified using Illumina HT12 microarrays and differential expression analysis (table S2). Similarly, genes potentially regulated by miR-20a were identified (table S3). Putative targets for miRNAs can be identified from their sequences, and we identified these using target prediction metadata collated in the miRecords database (http://mirecords.umn.edu/miRecords).

Genes that were differentially expressed as a result of knock-in or knock-down were compared against the predicted targets of the respective miRNA using *in silico* analysis. The resulting lists of genes were considered more likely to be directly affected by the changes in miRNA.

Gene expression data was used to identify gene pathways over-represented in the set of dysregulated genes in the transformed Jurkat cells using GeneGo maps module of Metacore (Genego, MI) (tables S4, S5). Translation (P<10^−4^ for miR-20a), cholesterol biosynthesis (P<10^−5^ for miR-20a, 10^−4^ for miR-17) and immune response pathways (P<10^−4^ for miR-17) were over-represented in the list of genes dysregulated by changes in miRNA expression.

We then tested if the genes regulated by the miRNAs miR-17 and 20a in Jurkat cells were also over-represented in MS whole blood. We previously reported, from a comparison of whole blood transcriptomes of 150 MS patients and controls, that T cell activation genes were markedly up-regulated in MS (P<10^−14^) [15]. We compared the gene expression profiles from transformed Jurkat cells against non-transformed cells. Putative miR-17 or miR-20a target genes were then compared with gene expression profiles from the MS whole blood samples to identify genes likely to be affected by these miRNAs.

**Figure 2:**
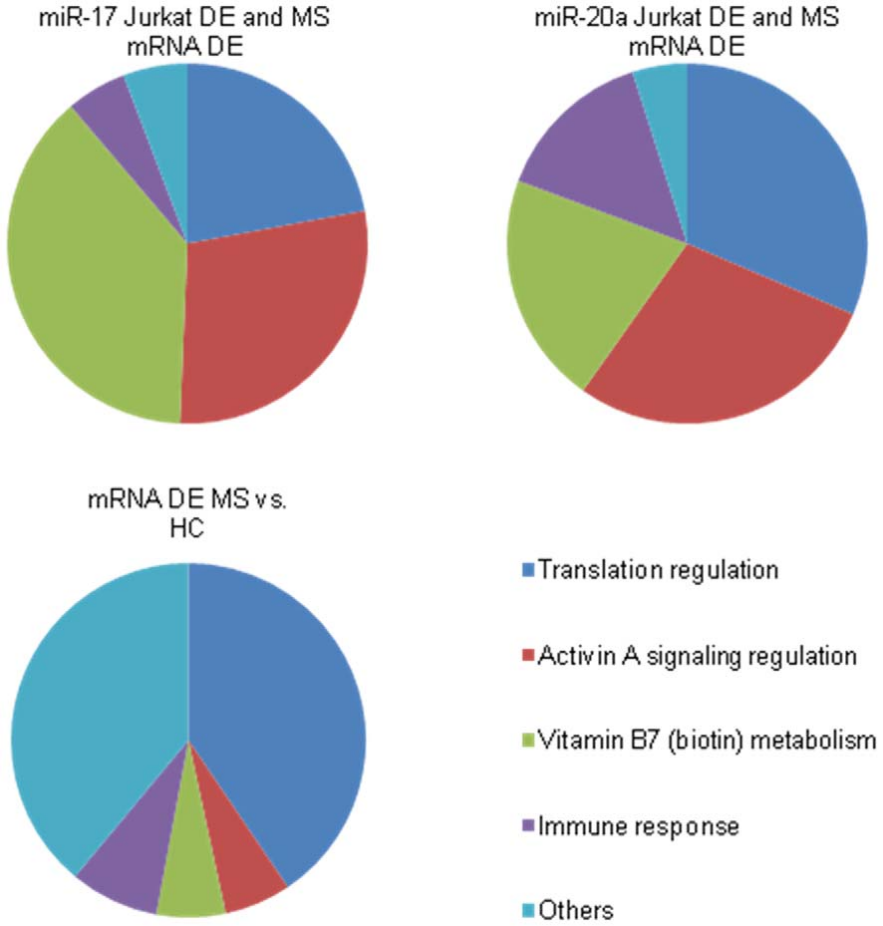
Gene networks dysregulated in MS are regulated by miR-17 and miR-20a miRNAs. The pathways enriched with genes both dysregulated in MS-whole blood and modulated in miR-17/20a transformed Jurkat cells were identified using Metacore (Tables S4 and S5). Most significant pathways are represented in the form of pie-charts where each slice represents −log_10_ of the p-value of that pathway as a proportion of the sum of the −log10 of the p-values of the over-represented pathways (P<0.05 for miR-17, p<0.001 for miR-20a and mRNA (MS vs. HC)). The p-values were determined by Metacore Pathway analysis based on the chi squared value for the expected compared to observed number of genes identified from that pathway in the list of dysregulated genes. (DE differential expression).

From just the mRNA and transformed Jurkat cell list, the pathways represented were very similar to the pathways over-represented in mRNA alone (Figure 2). Genes which were on all three lists were the most likely to be regulated by the miRNA in MS. From all 3 lists, transcription (P<10^−6^) and immune response pathways (P<10^−5^) were over-represented for miR-20a; and the Th17 immune response pathway (P<10^−3^) for miR-17.

## Discussion

Dysregulation of the immune system in MS is considered to be a fundamental aspect of disease initiation and development in this neurodegenerative disease. In this report we have investigated the miRNA transcriptome in a series of MS patients who were not on current immunomodulatory treatment. 59 clinically definite MS patients (mean age of 54.2 years), enriched for PP subtype (n = 18), with 24 RR and 17 SP were included in the analysis compared to 37 controls (mean age 48.0 years). The mean disease duration even for the relapsing remitting group was over 15 years. The selection of treatment naïve patients suggests that they were not a group with high disease activity. It could be argued that the significance of the findings of miR-17 and miR-20a being down regulated is underestimated compared to a more active northern European MS population. From the miRNA transcriptome comprising 733 miRNA species only one was up-regulated whereas 26 miRNAs were down-regulated with a false discovery rate less than one. From this data we focused on two miRNAs, miR-17 and miR-20a, as these were all under-expressed in all subtypes of MS and are known to be involved in the control of immune function [18].

Although miR-17 and miR-20a are encoded in the same cistron (miR-17-92), other miRNAs from this cistron were not under-expressed, and these two miRNAs were not identically expressed. There appears to be post-transcriptional regulation of the cistron miRNAs, allowing them to regulate different processes [19]. The translation pathway of lymphocytes is orchestrated by miR-17 and miR-20a [20]. Elevated expression of miR-17 and related miRNAs has been found to be associated with lymphoma and other cancers, and is probably important in maintaining an undifferentiated phenotype and resisting apoptosis [21], [22]. A reduction of miR-17-92 miRNAs observed in the present study, has also been associated with differentiation [23] and could potentially be involved in MS-associated T cell activation. We have previously demonstrated that mRNAs from these pathways were up-regulated in all clinical subtypes of MS [15]. In order to test whether these down-regulated miRNAs, miR-17 and 20a inhibit the expression of these T cell mRNAs we used knock in (with the miRNAs) and knock-down (with the antisense or siRNAs) assays of Jurkat cells, a model for T cells.

Jurkat cells are derived from a T cell lymphoma patient, and have traits of a partially differentiated T cell line [24], [25]. The transformed Jurkat cells were not stimulated, whereas the T cells in whole blood represent a myriad of subsets in various states of stimulation. However, as these miRNAs under-expressed in MS were known to increase transcription of genes involved with translation and immune response pathways, we expected they may also contribute to the pattern of gene dysregulation seen in MS whole blood mRNA. The set of genes down-regulated by miR-17 and miR-20a in transformed Jurkat cells overlap with the genes and the pathways they are predicted to control, suggesting these miRNAs contribute to the gene dysregulation observed in whole blood.

To our knowledge there have been only five other publications investigating the role of miRNA in MS [26]–[30], four of which focus on the immune system in MS and the other on active and inactive central nervous system lesions. Otagui *et al.* [29] focused more on up-regulation during relapse. As they only examined small number of patients, their qPCR did not reach statistical significance. Keller *et al.* [28] examined 20 RRMS patients on treatment and identified a number of miRNAs that were up or down regulated. The report by Du *et al.* [26] identified miR-326 as a major determinant of disease in Chinese MS but not neuromyelitis optica (an inflammatory demyelinating disorder thought to be mediated by antibodies targeting aquaporin 4 [31]). In our study we did not identify any statistically significant change in miR-326 between MS patients and controls. Furthermore, subgroup analysis confirmed this finding. Since we do not know why this miRNA was chosen for analysis by Du *et al.* [26] we are unable to put forward any functional explanation to describe our discrepant results apart from differences observed in MS patients from Asian or Caucasian origin.

The results of this investigation indicate that miR-17 and miR-20a are implicated in the development of MS. Of special interest is miR-17 which we found to be down regulated in peripheral blood. Interestingly, Lindberg *et al.* [30] also identified miR-17 as being associated with MS in CD4^+^ cells, but the relationship was in the opposite direction to what we observed. This is likely to be a result of a type 1 error given that only 8 patients and 10 controls were examined by Lindberg *et al.* in their initial miRNA expression analysis and only 15 patients and 10 controls in their confirmatory study. This in combination with our study design that included the three major subgroups of MS whereas the patients participating in the Lindberg report were diagnosed with relapsing remitting disease only could potentially explain the differences observed in miR-17 expression. In our analysis we have examined 59 cases against 37 controls thereby providing a more robust statistical analysis. Another possibility for the discrepancy is that we examined expression in whole blood collected in PAXgene RNA tubes, which stabilizes RNA on collection, and includes a high proportion of neutrophil RNA. One possibility is that miR-17 is very labile, and if it degraded in the 90 minutes or so longer it took Lindberg *et al* [30] to purify their miRNA - it could have degraded preferentially in the healthy control cells because of the different regulatory environment. Also, it may be that miR-17 is down-regulated in MS neutrophils cf controls, so that the net miR-17 we measured was less, even if it was more abundant in MS CD3 cells.

Even if the miRNAs under-expressed in MS were not directly contributing to the immune cell signature observed in MS whole blood, the excessive T cell activation signature seen in MS [15]–[17]; and other autoimmune diseases [32] suggest agents which can reduce this activity may be therapeutically beneficial. This study suggests, that the miRNAs identified here as under-expressed in MS, especially miR-17 and miR-20a, are regulators of genes involved in T cell activation.

## Methods

### Ethics Statement

Human Research Ethics Committees from the Sydney West Area Health Service, The Hunter New England Area Health Service and the Gold Coast Hospital approved this study. Written informed consent was given by all participants.

### Patients and sample collection

Peripheral blood samples were collected from MS patients and healthy controls in PAXgene™ Blood RNA tubes (Qiagen, Germany). Patients were recruited from Westmead Millennium Institute, Sydney (NSW), John Hunter Hospital, Newcastle (NSW) and Griffith University Gold Coast (QLD). Healthy controls, with no history of any autoimmune disease or any immunomodulatory therapy, were recruited from Westmead Millenium Institute, Sydney (NSW) and the John Hunter Hospital, Newcastle, (NSW). Patient and healthy controls samples were collected between 0900h and 1300h over the period of one year and collected in PAXgene tubes (PAXgene™ Blood RNA kit Qiagen, Hilden, Germany). Patients had received no immunomodulatory therapy within the last 3 months. MS diagnosis was according to Poser and McDonald criteria. The mean age of the MS patients was 54.2 years, and the controls 40.8 years. The mean age of the PPMS patients was 57.1 years; the mean age of the SPMS patients was 57.2 years and the mean age of the RRMS patients was 49.9 years (see Table S1).

### MiRNA expression analysis

#### RNA extraction

Patient and healthy control samples were processed concurrently to prevent batch effects. Frozen PAXgene blood RNA samples were thawed overnight at room temperature, centrifuged (4 minutes, 3,000g), and the supernatant discarded. The pellet was resuspended in 1ml TRIzol reagent (Invitrogen), briefly vortexed and homogenised. TRIzol RNA extraction was continued according to manufacturer's instructions. Briefly, 200µl chloroform was added, mixed thoroughly, incubated for 2 minutes at room temperature and centrifuged (15 minutes, 12,000g, 4°C). The aqueous phase containing the RNA was removed and added to 500µl isoproanol, incubated for 10 minutes at room temperature and centrifuged (10 minutes, 12,000g, 4°C). The supernatant was removed and the RNA pellet washed in 1ml 75% ethanol and centrifuged (5 minutes, 7,500g, 4°C) (repeated once). The supernatant was removed and the RNA resuspended in 20µl water.

The total RNA concentration was determined using RiboGreen (Ambion, TX) quantitation according to manufacturer's protocol.

#### Illumina miRNA SAM methods

MiRNA microarray assay using Illumina sentrix array matrix (SAM) was performed according to manufacturer's instructions (Illumina, CA) using 59 MS patients (24 RRMS, 17 SPMS and 18 PPMS) and 37 healthy controls. Briefly, 200ng total RNA was polyadenylated, reverse-transcribed and biotinylated. The cDNA was used in a second strand synthesis reaction, and universal amplification reaction involving the incorporation of a fluorescent marker. The labeled product was hybridised to the SAM and imaged using the Illumina BeadArray reader.

The microarray data was submitted to the gene expression omnibus (www.ncbi.nlm.nih.gov/geo/) under access number GSE21079.

#### Differential Expression Analysis

Data was quantile normalised by Illumina's BeadStudio V3. The differentially expressed miRNAs between MS and HC were identified using Significance Analysis of Microarray at a false discovery rate of less than 1%.

#### Real time PCR confirmation

Two miRNAs, miR-17 and miR-20a, were selected for quantitative PCR confirmation, using endogenous control U49. The sample cohort differs slightly from the cohort used in the microarray experiment (see table S1) due to small amount of RNA isolated from some samples, and the inclusion of extra samples which were not used on the 96 sample microarray. Final samples numbers were 57 MS (25 RRMS, 14 SPMS and 18 PPMS), and 34 healthy controls.

Reverse transcription of miRNA was performed on 11.35ng total RNA using the TaqMan miRNA Reverse Transcription Kit (Applied Biosystems), and pooled miRNA-specific primers. qPCR was performed using TaqMan miRNA assays (Applied Biosystems, CA) specific for each miRNA in triplicate for each sample. The sample was repeated if the standard deviation between triplicates was greater than 0.33.

The relative expression level was calculated using the comparative Ct method, and an unpaired, one-tailed t-test performed to test for significant difference between the entire MS cohort, as well as subtypes compared to the healthy controls.

### miRNA-perturbed gene expression analysis

#### Cell transfection

Jurkat cell cultures were maintained in suspension at 37°C with 5% CO_2_ and 90% humidity in RPMI 1640 supplemented with 10% (vol/vol) foetal calf serum and 2 mM L-glutamine. Synthetic miRNA (Sigma) or LNA modified antimiRs (Integrated DNA Technologies, CA) were delivered by electroporation (1µg oligonucleotide per 1×10^6^ cells) using a Nucleofector device and reagent V according to the manufacturer's instructions (Amaxa, Quantum Scientific, QLD), performed in triplicate. After 24 hours, cells were harvested by centrifugation and washed in PBS. Total RNA was then extracted from the cell pellets in TRIzol according to the manufacturer's instructions (Invitrogen, CA).

#### Whole genome gene expression analysis

500ng total RNA from each sample was biotinylated and amplified using Illumina TotalPrep RNA Amplification Kit (Ambion, TX) according to manufacturer's instructions. The cRNA yield was measured at using RiboGreen RNA quantitation kit (Invitrogen, CA) and 750ng of cRNA sample was hybridized on a human HT-12 expression beadchip (Illumina, CA) profiling 48,804 transcripts per sample. The chips were stained with streptavidin and scanned using an Illumina BeadArray Reader. Three biological replicates were performed for each transfection experiment.

#### Differential gene expression analysis

BeadStudio V3 was used to cubic spline normalise the data, with Quantile normalisation and baseline transformation to median of all samples performed in GeneSpring GX10. Gene expression profiles from miRNA and antimiRs electroporated Jurkat cells were generated and compared with profiles derived from untreated cells, and those electroporated with non-targeting control oligonucleotides using GeneSpring (unpaired t-test, p<0.05). Genes down regulated in response to the introduction of synthetic miRNA or up regulated in response to the introduction of the miRNA targeting anitmiR were cross-matched with the respective miRNA target genes predicted by a range of target algorithms supported by the miRecords target gene meta site (http://mirecords.umn.edu/miRecords/prediction_query.php). In order to capture a broad range of potential target sites, the conditions were set such that each target only need to satisfy the criteria for two out of a possible 11 different search algorithms. The predicted target genes found to be modulated in response to specific changes miRNA expression in electroporated cells were then examined to determine their potential for biological implications in the context of MS.

## Supporting Information

Table S1 Demographics of multiple sclerosis and control individuals. MS Multiple Sclerosis; RRMS relapsing remitting MS; SPMS secondary progressive MS; PPMS primary progressive MS; EDSS Expanded disability status scale
(0.06 MB PDF)

Table S2 Genes dysregulated in miR-17 knock-in and knock-down Jurkat transformants. DE - differential expression; MS - Multiple Sclerosis; red - up-regulated in MS; black - down-regulated in MS
(0.28 MB PDF)

Table S3 Genes dysregulated in miR-20a knock-in and knock-down Jurkat transformants. DE - differential expression; MS - Multiple Sclerosis; red - up-regulated in MS; black - down-regulated in MS
(0.37 MB PDF)

Table S4 Gene networks implicated in Multiple Sclerosis (MS) pathogenesis from miR-17 knock-in and -down experiments and from mRNA expression in whole blood.
(0.07 MB PDF)

Table S5 Gene networks implicated in Multiple Sclerosis (MS) pathogenesis from miR-20a knock-in and -down experiments and from mRNA expression in whole blood.
(0.04 MB PDF)

## References

[1] Hauser SL, Goodin DS, Fauci AS, Braunwald E, Kasper DL, Hauser SL, Longo DL (2008). Multiple Sclerosis and Other Demyelinating Diseases.. Harrison's Principles of Internal Medicine. 17 ed..

[2] Compston A, Coles A (2008). Multiple sclerosis.. Lancet.

[3] De Jager PL, Jia X, Wang J, de Bakker PIW, Ottoboni L (2009). Meta-analysis of genome scans and replication identify CD6, IRF8 and TNFRSF1A as new multiple sclerosis susceptibility loci.. Nat Genet.

[4] Hafler DA, Compston A, Sawcer S, Lander ES, Daly MJ (2007). Risk alleles for multiple sclerosis identified by a genomewide study.. N Engl J Med.

[5] ANZgene (2009). Genome-wide association study identifies new multiple sclerosis susceptibility loci on chromosomes 12 and 20.. Nat Genet.

[6] Baltimore D, Boldin MP, O'Connell RM, Rao DS, Taganov KD (2008). MicroRNAs: new regulators of immune cell development and function.. Nat Immunol.

[7] Xie X, Lu J, Kulbokas EJ, Golub TR, Mootha V (2005). Systematic discovery of regulatory motifs in human promoters and 3′ UTRs by comparison of several mammals.. Nature.

[8] Bartel DP (2004). MicroRNAs: genomics, biogenesis, mechanism, and function.. Cell.

[9] Sempere LF, Freemantle S, Pitha-Rowe I, Moss E, Dmitrovsky E (2004). Expression profiling of mammalian microRNAs uncovers a subset of brain-expressed microRNAs with possible roles in murine and human neuronal differentiation.. Genome Biol.

[10] Brown BD, Naldini L (2009). Exploiting and antagonizing microRNA regulation for therapeutic and experimental applications.. Nat Rev Genet.

[11] Wu H, Neilson JR, Kumar P, Manocha M, Shankar P (2007). miRNA profiling of naive, effector and memory CD8 T cells.. PLoS One.

[12] Liston A, Lu L-F, O'Carroll D, Tarakhovsky A, Rudensky AY (2008). Dicer-dependent microRNA pathway safeguards regulatory T cell function.. J Exp Med.

[13] Cobb BS, Hertweck A, Smith J, O'Connor E, Graf D (2006). A role for Dicer in immune regulation.. J Exp Med.

[14] Comi G (2009). Treatment of multiple sclerosis: role of natalizumab.. Neurol Sci.

[15] Gandhi KS, McKay FC, Cox M, Riveros C, Armstrong N The multiple sclerosis whole blood mRNA transcriptome and genetic associations indicate dysregulation of specific T cell pathways in pathogenesis.. Hum Mol Genet.

[16] Corvol JC, Pelletier D, Henry RG, Caillier SJ, Wang J (2008). Abrogation of T cell quiescence characterizes patients at high risk for multiple sclerosis after the initial neurological event.. Proc Natl Acad Sci U S A.

[17] Satoh J, Misawa T, Tabunoki H, Yamamura T (2008). Molecular network analysis of T-cell transcriptome suggests aberrant regulation of gene expression by NF-kappaB as a biomarker for relapse of multiple sclerosis.. Dis Markers.

[18] Tsitsiou E, Lindsay MA (2009). microRNAs and the immune response.. Curr Opin Pharmacol.

[19] Castellano L, Giamas G, Jacob J, Coombes RC, Lucchesi W (2009). The estrogen receptor-alpha-induced microRNA signature regulates itself and its transcriptional response.. Proc Natl Acad Sci U S A.

[20] O'Donnell KA, Wentzel EA, Zeller KI, Dang CV, Mendell JT (2005). c-Myc-regulated microRNAs modulate E2F1 expression.. Nature.

[21] Satoh J-I, Nakanishi M, Koike F, Miyake S, Yamamoto T (2005). Microarray analysis identifies an aberrant expression of apoptosis and DNA damage-regulatory genes in multiple sclerosis.. Neurobiology of Disease.

[22] Inomata M, Tagawa H, Guo YM, Kameoka Y, Takahashi N (2009). MicroRNA-17-92 down-regulates expression of distinct targets in different B-cell lymphoma subtypes.. Blood.

[23] Beveridge NJ, Tooney PA, Carroll AP, Tran N, Cairns MJ (2009). Down-regulation of miR-17 family expression in response to retinoic acid induced neuronal differentiation.. Cell Signal.

[24] Howe CJ, LaHair MM, Robinson PJ, Rodriguez-Mora O, McCubrey JA (2003). Models of anergy in the human Jurkat T cell line.. Assay Drug Dev Technol.

[25] Schneider U, Schwenk HU, Bornkamm G (1977). Characterization of EBV-genome negative “null” and “T” cell lines derived from children with acute lymphoblastic leukemia and leukemic transformed non-Hodgkin lymphoma.. Int J Cancer.

[26] Du C, Liu C, Kang J, Zhao G, Ye Z (2009). MicroRNA miR-326 regulates TH-17 differentiation and is associated with the pathogenesis of multiple sclerosis.. Nat Immunol.

[27] Junker A, Krumbholz M, Eisele S, Mohan H, Augstein F (2009). MicroRNA profiling of multiple sclerosis lesions identifies modulators of the regulatory protein CD47.. Brain.

[28] Keller A, Leidinger P, Lange J, Borries A, Schroers H (2009). Multiple sclerosis: microRNA expression profiles accurately differentiate patients with relapsing-remitting disease from healthy controls.. PLoS One.

[29] Otaegui D, Baranzini SE, Armananzas R, Calvo B, Munoz-Culla M (2009). Differential micro RNA expression in PBMC from multiple sclerosis patients.. PLoS One.

[30] Lindberg RLP, Hoffmann F, Mehling M, Kuhle J, Kappos L (2010). Altered expression of miR-17-5p in CD4+ lymphocytes of relapsing-remitting multiple sclerosis patients.. European Journal of Immunology.

[31] Weinshenker BG (2003). Neuromyelitis optica: what it is and what it might be.. Lancet.

[32] Podojil JR, Miller SD (2009). Molecular mechanisms of T-cell receptor and costimulatory molecule ligation/blockade in autoimmune disease therapy.. Immunol Rev.

