# Supplementary material for: MicroRNAs miR-17 and miR-20a Inhibit T Cell Activation Genes and Are Under-Expressed in MS Whole Blood
Source: PLoS One. 2010 Aug 11;5(8):e12132. doi: 10.1371/journal.pone.0012132 (PMC2920328; doi:10.1371/journal.pone.0012132)
Supplement: Table S1 — Demographics of multiple sclerosis and control individuals. MS Multiple Sclerosis; RRMS relapsing remitting MS; SPMS secondary progressive MS; PPMS primary progressive MS; EDSS Expanded disability status scale (0.06 MB PDF) [file pone.0012132.s001.pdf]

Table S1.
Demographics of multiple sclerosis and control individuals

		miRNA microarray							q RT PCR						
		number of samples	average age (years)	age range	average EDSS	EDSS range	average disease duration (years)	disease duration range (years)	number of samples	average age (years)	age range (years)	average EDSS	EDSS range	average disease duration (years)	disease duration range (years)
MS	Total	59	54.2	32 - 81	4.5	0 - 5.8	20.3	1 - 58	57	53.8	32 - 81	4.4	0 - 8.5	23.1	1 - 66
	Male	19	53.1	32 – 66	5.4	1.5 - 8.5	21.7	1 - 58	18	52.7	32 - 66	5.3	1.5 - 8.5	31.1	1 - 66
	Female	40	54.7	34 – 81	4.1	0 - 8.5	19.1	1 - 53	39	54.3	34 - 81	4.0	0 - 8.5	19.8	1 - 62
RRMS	Total	24	49.9	33 – 64	2.4	0 - 6.5	16.8	1 - 36	25	48.8	33 - 64	2.4	0 - 6.5	15.9	1 - 36
	Male	3	42.3	33 – 52	3.5	1.5 - 6	13.0	1 - 25	3	42.3	33 - 52	3.5	1.5 - 6	13.0	1 - 25
	Female	21	51.0	35 – 64	2.2	0 - 6.5	17.3	1 - 36	22	49.7	35 - 64	2.3	0 - 6.5	16.3	1 - 36
SPMS	Total	17	57.2	34 – 73	6.4	4 - 8.5	23.2	2 - 53	14	56.6	34 - 73	6.5	4 - 8.5	20.6	2 - 37
	Male	4	54.8	44 – 63	6.5	4 - 8.5	22.8	21 - 26	4	54.8	44 - 63	6.5	4 - 8.5	22.8	21 - 26
	Female	13	58.0	34 – 73	6.4	4 - 8.5	23.3	2 - 53	10	57.3	34 - 73	6.5	4 - 8.5	19.7	2 - 37
PPMS	Total	18	57.1	32 – 81	5.4	2 - 8	22.9	4 - 58	18	58.9	32 - 81	5.6	3.5 - 8	36.6	4 - 66
	Male	12	55.3	32 – 66	5.5	3.5 - 8	28.8	5 - 58	11	55.0	32 - 66	5.4	3.5 - 8	40.8	5 - 66
	Female	6	60.7	36 – 81	5.4	2 - 7.5	14.0	4 - 34	7	64.4	56 - 81	5.9	5 - 7	31.3	4 - 62
Control	Total	37	48.0	23 – 77	-	-	-	-	34	48.3	23 - 77	-	-	-	-
	Male	16	54.2	26 – 69	-	-	-	-	15	56.1	26 - 69	-	-	-	-
	Female	21	43.2	23 – 77	-	-	-	-	19	42.1	23 - 77	-	-	-	-

MS Multiple Sclerosis; RRMS relapsing remitting MS; SPMS secondary progressive MS; PPMS primary progressive MS; EDSS Expanded disability status scale
