# Supplementary material for: MicroRNAs miR-17 and miR-20a Inhibit T Cell Activation Genes and Are Under-Expressed in MS Whole Blood
Source: PLoS One. 2010 Aug 11;5(8):e12132. doi: 10.1371/journal.pone.0012132 (PMC2920328; doi:10.1371/journal.pone.0012132)
Supplement: Table S2 — Genes dysregulated in miR-17 knock-in and knock-down Jurkat transformants. DE - differential expression; MS - Multiple Sclerosis; red - up-regulated in MS; black - down-regulated in MS (0.28 MB PDF) [file pone.0012132.s002.pdf]

Table S2

Genes dysregulated in miR-17 knock-in and knock-down Jurkat transformants

miR-17 (Jurkat DE + predicted target genes of miRNA)	MS DE mRNA + miR-17 Jurkat DE	MS DE mRNA + miR-17 (Jurkat DE + Target)
MARCH5	C17orf39	C17orf39
SEPT7	CAP1	CAP1
AADAC	CASC3	CASC3
ABAT	CEBPB	CEBPB
ABCD3	DLST	FRAT1
ABHD13	DPF2	HLA-E
ABL1	FRAT1	IDS
ABTB1	FTL	ITGB5
ACAT2	HIST1H2BD	MXD1
ACSS2	HIST1H2BK	MYADM
ACTR10	HLA-E	NINJ1
ACVR2B	IDS	NUMB
ADAR	ITGB5	PRCP
ADM	MXD1	SLBP
ADM2	NUMB	TSC22D1
ADSSL1	PILRA	TXNIP
AGFG2	PRCP	UBAP1
AKAP13	PRSS7	UPF3B
ALDH3A2	SEC14L1	ZNF213
ALKBH1	SNX30	APOOL
AMDHD1	STAU1	ARMET
AMPD2	STK40	C6orf48
ANKS4B	STX11	CDKN2C
ANXA6	TSC22D1	COMMD3
AP4E1	TST	COQ5
APOBEC3F	TXNIP	DARS
APOOL	UBAP1	DUSP8
APPL2	UPF3B	IL23A
ARF6	ZNF213	MRPS22
ARFGEF2	ASF1B	WDR33
ARHGEF10	CDKN1B	ZNF740
ARID4B	CORO1A	BPGM
ARL4A	MYADM	PTPRCAP
ARL5B	NINJ1	RPL32
ARMET	SLBP	
ARNT2	APEX1	
ASL	APOOL	
ATCAY	ARMET	
ATF3	C19orf48	
ATF5	C6orf48	
ATP5SL	CCDC53	

ATP6V1H	CCDC84
ATPAF1	CCNB1IP1
ATRX	CDKN2C
ATXN7	CHPF
B3GAT1	CIP29
B3GNT1	COMMD3
BBS7	COQ5
BCL2A1	DARS
BCL2L11	DRG1
BMPR1A	DUSP8
BMPR2	EIF2B4
BPGM	EIF3D
BPTF	HEMGN
BRUNOL5	HYLS1
BTN1A1	IL23A
BZW1	LSM5
C10orf58	MED10
C11orf63	MRFAP1L1
C12orf44	MRPS21
C12orf48	MRPS22
C12orf49	NOSIP
C14orf138	NUP37
C16orf58	RIOK2
C16orf59	RPL17
C17orf39	RPLP0
C17orf48	STK16
C19orf59	STOML2
C1orf106	WDR33
C1orf107	WDR61
C1orf124	ZNF740
C1orf135	ZNHIT3
C1orf21	ATP5J
C1orf220	BPGM
C1orf9	CDC42
C1orf91	PRDX1
C1orf95	PTPRCAP
C20orf111	RPL13A
C2orf30	RPL32
C4orf3	TAF15
C4orf34	TCEAL4
C4orf49	UQCRFS1
C5orf41	
C5orf45	
C5orf51	
C6orf48	
C6orf62	

C7orf26
C7orf43
C7orf53
C9orf102
C9orf72
CACNB2
CACNB2
CACNB4
CADM2
CAMK2D
CAP1
CAPN3
CAPN3
CAPRIN2
CAPZA1
CASC1
CASC3
CAV3
CCDC109A
CCDC127
CCDC150
CCDC3
CCDC62
CCDC86
CCDC90B
CCL5
CCL8
CCND2
CCRK
CCS
CD14
CD163
CD38
CD5
CD82
CD83
CDC2L5
CDH26
CDK8
CDKN2C
CDYL2
CEBPB
CER1
CES3
CH25H
CHCHD4

CHIC1
CHIC2
CHRNB1
CLEC4A
CLSTN1
CLUAP1
COG3
COG5
COL2A1
COMMD3
COPS8
COQ10B
COQ5
COX11
CPEB4
CPZ
CRCP
CREB3L2
CRK
CRMP1
CRTAP
CRTC1
CRYZL1
CSNK2A2
CTAGE5
CTPS2
CUX2
CXCL12
CXorf1
CYTIP
DAG1
DARS
DCDC2
DCLRE1C
DDHD1
DDX17
DEDD
DENND5A
DGUOK
DGUOK
DHRS2
DHX57
DIABLO
DKK2
DLEC1
DLG5

DMD
DNAJB5
DNAJB6
DNAJB9
DNAJC27
DNALI1
DNASE2
DNMT3A
DNMT3A
DOCK4
DPY19L4
DRD2
DUOX1
DUSP8
DUT
DUXA
DYNC1I1
DYRK3
DZIP3
EBF1
EFHA2
EFTUD1
EHMT1
EIF1AX
EIF2B2
ELMO1
ELMOD3
EML1
EMP1
ENO3
ERO1L
ERO1LB
ESRRG
ETV3L
ETV5
EVI1
EXOC7
FADS1
FAM123A
FAM12B
FAM165B
FAM19A4
FAM24B
FAM38B2
FAM63A
FAM71E1

FAM82A2
FBLIM1
FBXO22
FBXO41
FBXW8
FCF1
FEN1
FLJ41603
FMNL3
FMO5
FNBP4
FOXD4L2
FOXO1
FOXP4
FRAT1
FUNDC1
GABARAPL1
GABRA1
GABRB2
GADD45B
GALK2
GAS8
GBF1
GBP6
GDAP1
GDF6
GGPS1
GIMAP2
GIMAP4
GLT25D2
GM2A
GNAO1
GNG7
GOT1
GPATCH8
GPR83
GPR89A
GRB10
GRB2
GRLF1
H1F0
HAUS1
HAUS2
HBEGF
HBP1
HDDC3

HES7
HIF3A
HINT3
HIP1
HLA-E
HMGA1
HMGCS1
HOMEZ
HOOK3
HOXC6
HPS4
HRH1
HSH2D
HTN1
HTR7
HVCN1
HYOU1
IDS
IFI44L
IFIT3
IFNA17
IFT140
IGF1
IGF2BP1
IGFBP5
IGSF3
IL23A
INA
ING1
ING4
INO80D
INSIG1
INSM2
IPO13
ISX
ITGB5
ITM2C
ITPR1
JAG2
JOSD1
KCNAB1
KCNH5
KCNH7
KCNJ10
KCNK3
KCNMA1

KCNMB2
KDM3B
KHDC1
KHK
KIAA0774
KIAA1370
KIF3B
KLC4
KLF10
KLHL2
KLHL25
KLHL28
KLK5
KLK7
KLRD1
KRBA2
L3MBTL4
LAMA3
LARP2
LATS2
LDLR
LEPREL1
LEPROTL1
LIAS
LIF
LILRB3
LIMA1
LIPH
LMBRD1
LRRC1
LSS
LYPD5
LYPD6
LYZ
MAGEA5
MAP2K3
MARVELD3
MATN3
MCFD2
MFAP3L
MFAP5
MLC1
MLF2
MMP2
MPHOSPH8
MPP5

MRAP2
MRGPRX3
MRPL4
MRPS22
MRRF
MST150
MTMR14
MTPAP
MTUS1
MUC17
MUDENG
MUT
MVD
MXD1
MYADM
MYO1E
MYOM2
NAIF1
NAP1L2
NAP5
NCAPG2
NCBP2
NCOA5
NDUFV3
NFATC2IP
NGFRAP1
NINJ1
NLRP3
NME6
NMNAT2
NMUR1
NOVA2
NRXN1
NSFL1C
NSL1
NUDT16
NUFIP1
NUMB
NUP54
OGT
OPTN
OR7D2
ORAOV1
ORC5L
OSM
OXCT1

PASD1
PASK
PAX8
PBOV1
PCDH10
PCDHGC3
PCDHGC3
PCGF6
PDCD6
PDE1A
PDGFRA
PEX13
PEX16
PEX19
PGBD5
PGM1
PHC3
PHF17
PHF21A
PHYHIP
PIWIL2
PKNOX1
PLCXD2
PLEKHO2
PLGLB1
PLS1
PMEPA1
PMM1
PNPLA3
POLDIP2
POLDIP3
POLE
PPARA
PPP1R1C
PPP1R3F
PPP2R2A
PPP3CA
PPP4R1
PPP4R2
PRCP
PREPL
PRKACB
PRKCE
PRNP
PRR16
PRRC1

PRSS16
PSD
PTBP2
PTEN
PTGER3
PTK2
PTP4A1
PTPRCAP
PUM1
PUS7L
RAB11FIP4
RAB14
RAB30
RAB3GAP1
RAB43
RALB
RANBP17
RASA1
RBAK
RBM16
RCN2
RCOR2
REL
REPS2
REXO1L1
RFC3
RFFL
RFFL
RG9MTD3
RGS3
RHBDD2
RIT1
RNASE6
RNF144B
RNF213
RNF215
RNF34
RNF8
ROBO1
RORA
RORA
RP4-691N24.1
RPL32
RPL32
RPS6KA2
RRM2

RUFY1
RUNDC3A
RUNX2
RYBP
S100PBP
SAMD7
SAR1A
SAV1
SBK1
SC5DL
SCAMP5
SCIN
SDAD1
SDC4
SDHC
SDSL
SEMA3A
SEMA4G
SEMG2
SERPINB3
SERPINE2
SETD2
SETD4
SFMBT1
SGK3
SGSM1
SH3BP5
SH3GLB1
SIDT1
SIRT6
SLBP
SLC16A7
SLC19A2
SLC22A8
SLC28A2
SLC2A9
SLC34A2
SLC43A3
SLC4A4
SLK
SMAD7
SMOX
SMYD2
SNCA
SND1
SNIP

SNTB2
SNX22
SNX7
SOBP
SOCS4
SOS2
SPATA2
SPCS2
SPRR2F
SRBD1
SRR
SS18
SSH1
SSTR2
SSU72
ST6GAL1
ST8SIA5
STAR
STARD10
STAT5A
STC2
STIM1
STYXL1
SUPT4H1
SYN1
SYN2
TAF12
TAGAP
TBC1D19
TBC1D2
TBC1D3C
TCF7L2
TCTEX1D1
TEK
TFE3
TFEB
TFPI2
TGIF2
TGOLN2
THYN1
TICAM2
TIPARP
TLR8
TMEM14C
TMEM167B
TMEM22

TMEM62
TMEM64
TMEM71
TMEM87A
TMEM9
TMEM98
TMEM9B
TMTC4
TMUB2
TNFAIP8L1
TNFRSF17
TNFSF14
TNFSF4
TOPORS
TP53INP1
TPD52
TPK1
TPM3
TPMT
TPP2
TRAFD1
TRAK1
TRHDE
TRIB3
TRIM4
TRNP1
TRPC1
TSC22D1
TSC22D1
TSEN34
TSPYL6
TXNIP
UBAP1
UBE2A
UBE2G2
UBL3
UBOX5
UGT2B15
UGT2B17
ULK3
UNC13C
UPF3B
UQCR
USP21
USP35
USP48

USP53
VANGL1
VAPB
VPS25
VPS33B
VPS45
WDR31
WDR33
WNK3
XBP1
XKR6
XKR8
XPC
XPR1
YWHAH
ZBTB41
ZBTB46
ZCCHC14
ZDHHC11
ZFAND3
ZFP82
ZHX2
ZKSCAN1
ZMAT5
ZNF177
ZNF211
ZNF213
ZNF256
ZNF323
ZNF395
ZNF419
ZNF517
ZNF546
ZNF615
ZNF620
ZNF680
ZNF695
ZNF740
ZNF750
ZNF761
ZNF773
ZNF823
ZSWIM4
ZZZ3

DE - differential expression; MS - Multiple Sclerosis; red – up-regulated in MS; black – down-regulated in MS
