# Supplementary material for: MicroRNAs miR-17 and miR-20a Inhibit T Cell Activation Genes and Are Under-Expressed in MS Whole Blood
Source: PLoS One. 2010 Aug 11;5(8):e12132. doi: 10.1371/journal.pone.0012132 (PMC2920328; doi:10.1371/journal.pone.0012132)
Supplement: Table S3 — Genes dysregulated in miR-20a knock-in and knock-down Jurkat transformants. DE - differential expression; MS - Multiple Sclerosis; red - up-regulated in MS; black - down-regulated in MS (0.37 MB PDF) [file pone.0012132.s003.pdf]

Table S3

Genes dysregulated in miR-20a knock-in and knock-down Jurkat transformants

miR-20a (Jurkat DE + predicted target genes of miRNA)	MS DE mRNA + miR-20a Jurkat DE	MS DE mRNA + miR-20a (Jurkat DE + Target)
SEPT2	CASC3	ACTR2
SEPT2	CEBPB	ARHGAP30
SEPT7	CREBBP	BID
MARCH8	DPF2	BMP2K
AASDHPPT	FLI1	CASC3
ABAT	FTL	CEBPB
ABCC10	H3F3A	CREBBP
ABCD3	HIST1H2BD	EIF4EBP2
ABTB1	HIST1H2BK	H3F3A
ACAD11	IDS	IDS
ACADM	IFNA7	IFNA7
ACAT2	LIMS1	KRAS
ACTR10	NUAK2	LPCAT1
ACTR1A	OSBPL2	MAP4K4
ACTR2	PCTP	OSBPL2
ADAM17	PRSS7	PCTP
ADAMTSL2	RASSF2	RASSF2
ADM2	RHOB	RXRA
ADRA2B	RXRA	STK40
AGK	STK40	TRIOBP
AHNAK	TRIOBP	TSC22D1
AIPL1	UBE2D3	ZFP36
AKAP13	WIPI1	ACAD11
AKIRIN1	ZFP36	CDKN2C
ALDOC	ACTB	COQ5
ALG9	ACTR2	IL23A
ALKBH1	ARHGAP30	MFHAS1
ALPK2	ARMET	NAPB
AMACR	BID	WDR33
AMPD2	BMP2K	C13orf27
ANGPT1	EIF4EBP2	C20orf20
ANKRD40	ETS2	
ANXA13	HLA-DQB1	
AP2M1	KRAS	
AP3M1	LPCAT1	
ARAP2	MAP4K4	
ARCN1	SLBP	
ARHGAP30	SNX15	
ARHGEF9	TSC22D1	
ARIH2	ACAD11	
ARL4A	BOLA3	
ARL6IP5	BXDC1	

ARL6IP6	C6orf48
ARMC10	CCDC53
ARMC2	CCDC59
ARMCX1	CCNB1IP1
ARPP-21	CDKN2C
ARRB2	COQ5
ARRDC1	DRG1
ARSD	EIF2B4
ASL	ERP27
ASPH	HEMGN
ASS1	HSPE1
ATAD3B	IL23A
ATF5	LGALS1
ATG12	MCART1
ATP1B4	MFHAS1
BAGE	MRPS21
BBS7	NAPB
BCL2L11	NAT5
BECN1	NUCKS1
BID	NUP37
BMF	PIGF
BMP2K	RIOK2
BMPR1A	RPL12
BRP44L	RPL24
BTBD7	RPL36AL
C10orf46	RPL39
C10orf47	SFRS16
C11orf63	STK16
C12orf44	TIMM23
C12orf48	TMEM126A
C12orf66	WDR33
C12orf67	ANXA1
C12orf68	C13orf27
C13orf27	C20orf20
C14orf102	C21orf33
C14orf147	C22orf28
C15orf44	EIF3E
C15orf54	HIST1H4C
C16orf58	KLRB1
C17orf37	MRPL55
C1orf161	NAE1
C1orf21	NME1
C1orf52	RPL11
C1orf95	RPL13A
C20orf111	RPL41
C20orf117	RPL6

C20orf20	RPS15A
C21orf34	RPS27
C2orf66	RPS27A
C3orf15	RPS29
C4orf3	TAF15
C4orf30	
C4orf32	
C4orf34	
C4orf49	
C5AR1	
C5orf41	
C5orf51	
C6orf162	
C6orf182	
C6orf224	
C7orf41	
C7orf43	
C7orf53	
C8orf33	
C9orf102	
C9orf47	
C9orf97	
CACNA1E	
CACNA2D3	
CADM2	
CAMK2N1	
CAMKK1	
CAPN3	
CASC1	
CASC3	
CASP7	
CBFA2T2	
CBFA2T3	
CBL	
CC2D1A	
CCDC112	
CCDC132	
CCDC3	
CCDC52	
CCDC62	
CCDC86	
CCDC90B	
CCND3	
CCNG1	
CCNL1	
CCRK	

CCRL1
CD163
CD164
CD24
CD36
CD5
CD83
CDC2L5
CDC42SE2
CDH26
CDK2
CDK8
CDKN2C
CDT1
CEBPB
CEBPD
CES3
CHCHD7
CHD6
CHIC1
CHIC2
CHM
CHN2
CHRDL1
CHRNB1
CLCC1
CLDN16
CLDN18
CLDND1
CLEC2B
CLEC4A
CMKLR1
CMTM3
CMTM4
CNOT6
COBL
COG3
COL11A1
COL13A1
COL17A1
COL2A1
COMMD5
COPA
COPS2
COPS7A
COPS8

COQ10B
COQ5
CORO2B
CPA4
CPEB3
CR1
CREBBP
CRISPLD2
CRK
CRMP1
CSNK1E
CSNK2A2
CST9L
CSTF1
CTDSPL
CTSL1
CUGBP2
CUL4A
CXorf38
CYP19A1
CYP26B1
CYP4F3
CYP4X1
DACH1
DAG1
DAND5
DAZL
DBN1
DCDC2
DCHS1
DCLRE1C
DDR2
DDX47
DECR2
DEDD
DENND5A
DGUOK
DHCR24
DHRS2
DHX35
DHX9
DIAPH2
DICER1
DIP2B
DKK2
DLEC1

DNA2
DNAJB5
DNAJB7
DNAJB9
DNAJC27
DNAJC30
DNALI1
DNASE2
DOCK3
DPP3
DPY19L4
DPYD
DPYSL5
DSEL
DTNA
DTX4
DUOX1
DUSP10
DUSP10
DUSP4
DUSP6
DYNC1I1
DZIP3
ECHDC1
ECOP
EDA
EDNRB
EEF1A1
EFHC2
EFNA4
EFTUD1
EIF1AX
EIF2B2
EIF4A2
EIF4B
EIF4EBP2
EIF4H
EMR2
ENDOD1
ENPP6
ENTPD1
EPB41L2
ERBB2
ERO1L
ERO1LB
ERP29

ERP44
ESR1
ETS1
ETV5
EVI1
EXD1
EZH2
FADS1
FAM110A
FAM129A
FAM12B
FAM175A
FAM177A1
FAM38B2
FAM62A
FAM65A
FAM81A
FAM83C
FBXO11
FBXO41
FBXW7
FCGR3B
FEN1
FGF2
FGFR1
FKBP14
FLJ20184
FMNL3
FNTB
FOXK1
FOXP4
FOXRED2
FRMD6
FUT2
FUT4
FXN
FXR1
G3BP2
GABPA
GALK2
GALNT12
GALR2
GAS8
GATA3
GATAD2A
GBF1

GCOM1
GDA
GDF6
GFPT1
GGA1
GHRH
GIMAP4
GIPC1
GLIPR2
GM2A
GNE
GORAB
GPC3
GPM6A
GPR135
GPR81
GPR83
GRLF1
GRM7
GTF2H1
GTPBP1
H1F0
H3F3A
HADH
HARS
HBEGF
HDAC9
HEMK1
HERC6
HERPUD1
HERPUD2
HIAT1
HIST1H2AG
HLA-A
HLA-DRA
HMGB1
HMGCS1
HMGXB4
HNRNPC
HNRNPF
HNRNPR
HORMAD2
HOXC6
HOXD13
HP1BP3
HPRT1

HSD17B1
HSD17B6
HSPA8
HSPB3
HSPD1
HTN1
HVCN1
HYOU1
HYPK
ICA1L
IDH1
IDI2
IDS
IFNA17
IFNA7
IFT81
IGF2BP1
IL23A
IL8
ILF3
IMPA1
IMPDH1
INCENP
ING2
INPP5F
INSIG1
INSM2
IP6K1
IQGAP2
IRF8
IRF9
ISOC1
ITGA2
ITIH2
ITIH5
ITM2C
JTB
KANK1
KBTBD3
KCMF1
KCNH4
KCNH5
KCNMB2
KCNMB2
KCNQ1
KIAA0232

KIAA1024
KIAA1370
KIAA1715
KIAA1804
KIF20B
KIF21B
KIF5B
KLF10
KLHL24
KLHL25
KLHL28
KRAS
KRIT1
KRIT1
KRT10
LAIR1
LBR
LCORL
LDLR
LDLRAP1
LEPROTL1
LIF
LIPH
LLGL1
LMNB1
LOC153328
LOC643684
LPCAT1
LPIN1
LRRC1
LRRC31
LRRC40
LRRC47
LRRN2
LSS
LY6H
LYST
LYZ
MAEA
MAN1C1
MANBA
MAP4K4
MAPT
MAPT
MASP1
MCM4

MCM7
MDH1B
MED23
MEF2D
MEST
MFGE8
MFHAS1
MFN1
MFSD11
MGLL
MIER1
MLF2
MMAB
MMGT1
MORF4L2
MPP7
MRE11A
MRPL15
MRPL47
MST150
MSTO1
MTFR1
MUC17
MUDENG
MUT
MVD
MXRA7
MYH9
MYO18A
MYOM1
MYOM2
MYPN
NAP5
NAPB
NAT12
NBPF14
NCBP2
NDFIP2
NELF
NFAT5
NFATC1
NFATC2IP
NFYA
NHLRC3
NIP30
NMNAT2

NMT1
NONO
NOP2
NOTCH1
NOVA2
NP
NPY5R
NQO1
NTM
NUDT21
NUDT9
NUFIP1
NUP153
NUP54
NUPL1
OAZ1
OGN
OPN3
OPTN
ORC5L
OSBPL2
OSBPL6
OSTCL
OTUD6B
OTUD7A
PANK1
PAPD5
PAPPA
PAX2
PBOV1
PCDH11X
PCDHA6
PCDHA6
PCF11
PCGF6
PCTP
PDCD6
PDE1A
PDS5B
PDZD11
PEG3
PFN2
PGM2L1
PHACTR4
PHF17
PHF21A

PHKA1
PHYHIP
PI4K2B
PIF1
PIGA
PIGH
PIGY
PIK3IP1
PIK3R1
PIP4K2A
PIP4K2C
PISD
PITPNB
PLA2G12B
PLK4
PNMAL1
PODXL
POLDIP2
POLE
POLR3H
POU3F2
PPAP2B
PPM1B
PPM1K
PPP1R3B
PPP1R3F
PPP1R9B
PPP2R2C
PPP2R3A
PPP4R4
PPTC7
PRDM1
PRDM16
PRDM2
PRDM8
PRDM9
PRDX3
PRKAR1A
PRKAR1A
PRKCB
PRKDC
PRNP
PROK2
PRR15
PRR16
PRRG4

PRX
PSAT1
PSD3
PSD3
PSMD5
PTGER3
PTN
PTPRO
PUM1
PUS7L
PVRL2
RAB11A
RAB11FIP4
RAB27A
RAB35
RAB37
RAB3IP
RAB43
RABGEF1
RAD21
RAD23B
RAD54B
RALB
RAN
RANBP17
RAPGEF1
RARA
RARRES1
RASSF2
RB1
RBBP7
RBM12
RBM16
RCBTB1
RCOR2
RDBP
REM2
RFC2
RFFL
RFFL
RFXAP
RG9MTD3
RGS17
RHOBTB3
RHOT1
RIMS3

RNASE6
RNASEL
RNF19A
RNF215
RNF8
RNMTL1
ROBO1
RP2
RP6-213H19.1
RPA1
RPL18
RPP30
RPS6KA1
RPS6KA2
RRAS2
RTN1
RUFY1
RUNDC3A
RXRA
RYK
SAFB2
SAMD7
SAR1A
SC4MOL
SC5DL
SC65
SCAMP1
SCARB2
SCD
SCGB2A1
SCN9A
SCRIB
SCRN3
SCYL2
SDAD1
SDC4
SDSL
SEC23A
SEC61A2
SEL1L
SELI
SELL
SELM
SELT
SEMA4G
SEPHS1

SERPINB9
SERPINE2
SETD2
SF1
SFRS3
SGK269
SGOL1
SHISA2
SIRT5
SKAP2
SLC1A3
SLC22A2
SLC22A8
SLC25A10
SLC25A24
SLC25A34
SLC25A38
SLC26A1
SLC26A7
SLC27A6
SLC2A6
SLC2A9
SLC35B1
SLC37A1
SLC39A14
SLC48A1
SLC4A7
SLC7A1
SLC7A11
SLC7A5
SLMO2
SMAD6
SMCHD1
SMOC1
SMOX
SNAPIN
SNIP
SNN
SNRPD3
SNTB2
SNW1
SORBS2
SORD
SOX17
SOX7
SP1

SPCS3
SPINK7
SPIRE1
SPNS1
SPRED2
SPRN
SPRY4
SPSB4
SPTLC1
SR140
SRGAP2
SRR
SSR1
SSTR2
ST8SIA5
STAG1
STAG2
STAU2
STBD1
STIM1
STK40
STMN1
STX16
STYXL1
SURF6
SUV420H1
SYN1
SYNPO2L
SYT13
SYT15
SYVN1
TADA1L
TAF12
TAX1BP1
TBC1D17
TBC1D19
TBC1D2
TCEA1
TCEAL1
TCERG1
TCF20
TCF7L2
TCHH
TCTEX1D1
TDG
TDP1

TEC
TECPR1
TFG
TGFBR1
THAP10
THAP8
THSD4
THUMPD1
TIAM2
TICAM2
TIMP2
TIPIN
TM4SF19
TMEM106B
TMEM128
TMEM135
TMEM151A
TMEM168
TMEM188
TMEM20
TMEM214
TMEM217
TMPO
TMSB4X
TMTC4
TMX1
TNFAIP8
TNFRSF1A
TNFSF14
TNPO3
TOM1
TPD52
TPD52L2
TPD52L2
TPK1
TPM3
TPRG1
TRAF7
TRAPPC2
TRDMT1
TRIB3
TRIM58
TRIM68
TRIOBP
TRIP11
TRNP1

TRPC1
TSC22D1
TSC22D1
TSEN15
TSLP
TSPAN17
TSPYL4
TTC39A
TTN
TUBGCP4
TXLNB
UBC
UBE2D2
UBE2E2
UBE2K
UBE2L3
UBQLN1
UBTF
UBXN4
UGT2B15
UGT2B17
UNC13A
UNKL
UQCR
USP2
USP33
USP35
USP48
UTP14A
VANGL1
VASH1
VAT1L
VAV3
VEGFA
VENTX
VKORC1L1
VPS13B
VPS41
WDR1
WDR16
WDR31
WDR33
WHSC1L1
WNK3
WWOX
XBP1

XBP1
XKR4
XPR1
XRN2
YIPF2
ZBTB7A
ZC3H13
ZC3H14
ZDHHC11
ZDHHC4
ZFAND3
ZFAT
ZFP36
ZFYVE9
ZKSCAN1
ZMAT3
ZNF177
ZNF211
ZNF292
ZNF317
ZNF323
ZNF329
ZNF37A
ZNF468
ZNF491
ZNF510
ZNF530
ZNF584
ZNF587
ZNF607
ZNF626
ZNF669
ZNF672
ZNF681
ZNF682
ZNF691
ZNF695
ZNF750
ZNF761
ZNF766
ZNF800
ZNF823
ZRANB2
ZSCAN10

DE - differential expression; MS - Multiple Sclerosis; red – up-regulated in MS; black – down-regulated in MS
