# Supplementary material for: MicroRNAs miR-17 and miR-20a Inhibit T Cell Activation Genes and Are Under-Expressed in MS Whole Blood
Source: PLoS One. 2010 Aug 11;5(8):e12132. doi: 10.1371/journal.pone.0012132 (PMC2920328; doi:10.1371/journal.pone.0012132)
Supplement: Table S4 — Gene networks implicated in Multiple Sclerosis (MS) pathogenesis from miR-17 knock-in and -down experiments and from mRNA expression in whole blood. (0.07 MB PDF) [file pone.0012132.s004.pdf]

Table S4

Gene networks implicated in Multiple Sclerosis (MS) pathogenesis from miR-17 knock-in and -down experiments and from mRNA expression in whole blood

Pathway	P Value	No. Genes Represented	No. Genes on Pathway	No. Genes in miRNA list
**miR-17 Jurkat differential expression, miR-17 target genes, MS mRNA differential expression**				**34**
Immune response_IL-17 signalling pathways	7.82E-03	2	205	
**miR-17 Jurkat differential expression, MS mRNA differential expression**				**82**
Vitamin B7 (biotin) metabolism	3.78E-10	7	99	
Signal transduction_Activin A signalling regulation	8.54E-08	7	216	
Translation _Regulation of translation initiation	3.48E-06	7	376	
Translation_(L)-selenoaminoacids incorporation in proteins during translation	3.11E-04	5	349	
**miR-17 Jurkat differential expression, miR-17 target genes**				**683**
Development_Transactivation of PDGFR in non-neuronal cells by Dopamine D2 receptor	3.64E-04	8	143	
Development_A2B receptor: action via G-protein alpha s	3.80E-04	9	181	
Aspartate and asparagine metabolism	9.18E-04	5	62	
Chemotaxis_CXCR4 signalling pathway	9.35E-04	8	165	
Cholesterol Biosynthesis	9.88E-04	5	63	
Membrane-bound ESR1: interaction with G-proteins signalling	1.34E-03	9	216	
Development_Angiopoietin - Tie2 signalling	1.55E-03	7	139	
Development_A3 receptor signalling	1.71E-03	9	224	
Transcription_CREB pathway	2.90E-03	10	289	
Development_IGF-1 receptor signalling	3.32E-03	8	202	
Cytoskeleton remodeling_FAK signalling	3.42E-03	8	203	
HIV-1 signalling via CCR5 in macrophages and T lymphocytes	3.57E-03	6	121	
Development_EGFR signalling pathway	3.96E-03	8	208	
Immune response_Role of integrins in NK cells cytotoxicity	4.70E-03	6	128	
Thiamine metabolism	4.93E-03	2	9	
**miR-17 Jurkat differential expression**				**2187**
Development_Flt3 signalling	1.47E-04	14	157	
Development_A2B receptor: action via G-protein alpha s	1.95E-04	15	181	
Immune response_CD137 signalling in immune cell	1.98E-04	11	106	
Development_EGFR signalling pathway	2.84E-04	16	208	
Apoptosis and survival_Endoplasmic reticulum stress response pathway	1.14E-03	13	171	
Cholesterol Biosynthesis	1.89E-03	7	63	
Development_Transactivation of PDGFR in non-neuronal cells by Dopamine D2 receptor	2.41E-03	11	143	
Immune response_Role of DAP12 receptors in NK cells	3.35E-03	12	171	
G-protein signaling_Ras family GTPases in kinase cascades (scheme)	3.81E-03	8	90	
