# Supplementary material for: MicroRNAs miR-17 and miR-20a Inhibit T Cell Activation Genes and Are Under-Expressed in MS Whole Blood
Source: PLoS One. 2010 Aug 11;5(8):e12132. doi: 10.1371/journal.pone.0012132 (PMC2920328; doi:10.1371/journal.pone.0012132)
Supplement: Table S5 — Gene networks implicated in Multiple Sclerosis (MS) pathogenesis from miR-20a knock-in and -down experiments and from mRNA expression in whole blood. (0.04 MB PDF) [file pone.0012132.s005.pdf]

Table S5

Gene networks implicated in Multiple Sclerosis (MS) pathogenesis from miR-20a knock-in and -down experiments and from mRNA expression in whole blood

Pathway	P Value	No. Genes Represented	No. Genes on Pathway	No. Genes in miRNA list
**miR-20a Jurkat differential expression, miR-20a target genes, and MS mRNA differential expression**				**31**
Transcription_Sin3 and NuRD in transcription regulation	3.11E-06	4	150	
Immune response_Antiviral actions of Interferons	3.46E-06	4	154	
Immune response_TLR3 and TLR4 induce TICAM1-specific signaling pathway	2.85E-05	3	88	
Immune response_IFN alpha/beta signaling pathway	3.36E-05	3	93	
Immune response_Innate immunity response to RNA viral infection	7.92E-05	3	124	
Signal transduction_Activin A signaling regulation	4.06E-04	3	216	
G-protein signaling_Ras family GTPases in kinase cascades (scheme)	1.66E-03	2	90	
Cell cycle_Chromosome condensation in prometaphase	1.84E-03	2	95	
Apoptosis and survival_Cytoplasmic/mitochondrial transport of proapoptotic proteins Bid, Bmf and Bim	1.92E-03	2	97	
Cell cycle_Sister chromatid cohesion	2.08E-03	2	101	
Regulation of lipid metabolism_Regulation of lipid metabolism via LXR, NF-Y and SREBP	2.25E-03	2	105	
Development_Glucocorticoid receptor signaling	2.78E-03	2	117	
Transcription_Ligand-dependent activation of the ESR1/SP pathway	3.62E-03	2	134	
Transcription_Role of heterochromatin protein 1 (HP1) family in transcriptional silencing	3.67E-03	2	135	
Cell cycle_Initiation of mitosis	4.00E-03	2	141	
Cell cycle_Role of Nek in cell cycle regulation	5.24E-03	2	162	
**miR-20a Jurkat differential expression and MS mRNA differential expression**				**93**
Translation _Regulation of translation initiation	6.16E-14	14	376	
Signal transduction_Activin A signaling regulation	1.15E-12	11	216	
Translation_(L)-selenoaminoacids incorporation in proteins during translation	1.05E-11	12	349	
Vitamin B7 (biotin) metabolism	1.69E-09	7	99	
Immune response_Antiviral actions of Interferons	8.89E-07	6	154	
Transcription_Sin3 and NuRD in transcription regulation	1.60E-05	5	150	
Immune response_TLR3 and TLR4 induce TICAM1-specific signaling pathway	8.28E-04	3	88	
Immune response_IFN alpha/beta signaling pathway	9.72E-04	3	93	
Immune response_Innate immunity response to RNA viral infection	2.22E-03	3	124	
Transcription_Ligand-dependent activation of the ESR1/SP pathway	2.76E-03	3	134	
Immune response _Immunological synapse formation	3.32E-03	4	294	

Development_NOTCH1-mediated pathway for NF-KB activity modulation	5.03E-03	3	166	
Development_Notch Signaling Pathway	6.10E-03	3	178	
Transport_Macropinocytosis regulation by growth factors	8.50E-03	3	201	
**miR-20a Jurkat differential expression and miR-20a target genes**				**914**
Cholesterol Biosynthesis	7.23E-05	7	63	
Regulation of lipid metabolism_RXR-dependent regulation of lipid metabolism via PPAR, RAR and VDR	7.10E-04	6	66	
DNA damage_NHEJ mechanisms of DSBs repair	1.05E-03	6	71	
Transcription_Ligand-dependent activation of the ESR1/SP pathway	1.61E-03	8	134	
Regulation of lipid metabolism_Regulation of lipid metabolism via LXR, NF-Y and SREBP	1.66E-03	7	105	
Cell cycle_Start of DNA replication in early S phase	4.04E-03	7	123	
Apoptosis and survival_Cytoplasmic/mitochondrial transport of proapoptotic proteins Bid, Bmf and Bim	5.07E-03	6	97	
**miR-20a Jurkat differential expression**				**2741**
Cholesterol Biosynthesis	1.14E-05	11	63	
Translation _Regulation of translation initiation	1.90E-04	28	376	
Regulation of lipid metabolism_Regulation of lipid metabolism via LXR, NF-Y and SREBP	1.21E-03	11	105	
Translation_Insulin regulation of translation	2.21E-03	14	165	
Cell cycle_Spindle assembly and chromosome separation	2.56E-03	19	262	
Translation_(L)-selenoaminoacids incorporation in proteins during translation	3.30E-03	23	349	
Development_Flt3 signalling	3.84E-03	13	157	
